# Volatile Organic Compounds of *Bacillus velezensis* GJ-7 against *Meloidogyne hapla* through Multiple Prevention and Control Modes

**DOI:** 10.3390/molecules28073182

**Published:** 2023-04-03

**Authors:** Wentao Wu, Yuanling Zeng, Xirui Yan, Zhuhua Wang, Liwei Guo, Youyong Zhu, Yang Wang, Xiahong He

**Affiliations:** 1 Key Laboratory of Agricultural Biodiversity and Pest Control, College of Plant Protection, Yunnan Agricultural University, Kunming 650201, China; wentao_wu121@163.com (W.W.); zengyl311@163.com (Y.Z.); yan784534151@163.com (X.Y.); wzhuhua@126.com (Z.W.); g87l12w12@163.com (L.G.); yppl@public.km.yn.cn (Y.Z.); 2 Key Laboratory of Forest Resources Conservation and Utilization in the Southwest Mountains of China Ministry of Education, Southwest Forestry University, Kunming 650224, China

**Keywords:** *Bacillus velezensis* GJ-7, *Meloidogyne hapla*, solid-phase micro-extraction gas chromatography-mass spectrometry, volatile organic compounds (VOCs), biological control

## Abstract

The *Bacillus velezensis* GJ-7 strain isolated from the rhizosphere soil of *Panax notoginseng* showed high nematicidal activity and therefore has been considered a biological control agent that could act against the root-knot nematode *Meloidogyne hapla*. However, little was known about whether the GJ-7 strain could produce volatile organic compounds (VOCs) that were effective in biocontrol against *M. hapla*. In this study, we evaluated the nematicidal activity of VOCs produced by the fermentation of GJ-7 in three-compartment Petri dishes. The results revealed that the mortality rates of *M. hapla* J2s were 85% at 24 h and 97.1% at 48 h after treatment with the VOCs produced during GJ-7 fermentation. Subsequently, the VOCs produced by the GJ-7 strain were identified through solid-phase micro-extraction gas chromatography mass spectrometry (SPME-GC/MS). Six characteristic VOCs from the GJ-7 strain fermentation broth were identified, including 3-methyl-1-butanol, 3-methyl-2-pentanone, 5-methyl-2-hexanone, 2-heptanone, 2,5-dimethylpyrazine, and 6-methyl-2-heptanone. The in vitro experimental results from 24-well culture plates showed that the six volatiles had direct-contact nematicidal activity against *M. hapla* J2s and inhibition activity against egg hatching. In addition, 3-methyl-1-butanol and 2-heptanone showed significant fumigation effects on *M. hapla* J2s and eggs. Furthermore, all six of the VOCs repelled *M. hapla* J2 juveniles in 2% water agar Petri plates. The above data suggested that the VOCs of *B. velezensis* GJ-7 acted against *M. hapla* through multiple prevention and control modes (including direct-contact nematicidal activity, fumigant activity, and repellent activity), and therefore could be considered as potential biocontrol agents against root-knot nematodes.

## 1. Introduction

Plant parasitic nematodes (PPNs) cause severe damage to a wide range of crops, including agriculture crops, vegetables and even medicinal plants [1,2,3]. The root-knot nematode (*Meloidogyne* spp.) is one of the most important obligate plant parasitic nematode species; infections by this species induce root galling, prevent plant growth, and cause complex diseases in synergy with other soil-borne pathogens [4,5]. PPNs cause losses of more than USD100–150 billion worldwide every year, more than half of which are due to *Meloidogyne* spp. [3,6,7,8]. In the past, the primary method of controlling plant nematodes was to use chemical nematicides [9]. However, these chemicals are toxic; have negative side effects on the environment, animals, and humans; and have been restricted in many countries [10,11,12]. In addition, the crop rotation and selection of resistant varieties can also control PPNs, but the effectiveness of these methods is limited [13]. Therefore, new, effective and environmentally friendly biological control options are urgently needed [11].

As can be seen from previous studies, the use of biological control agents to control PPNs are gaining popularity [12,14]. Soil microorganisms are important biological control resources, and metabolites produced by these microorganisms are effective against several pathogenic bacteria, insects and nematodes [15]. As for microbial metabolites, volatile organic compounds (VOCs) have attracted research attention in recent years due to their strong nematicidal activity [16,17]. The greatest advantages of VOCs are their good dispersibility and penetration into the soil. Additionally, the application of VOCs in agricultural practice could be both economically affordable and less toxic to humans than conventional nematicides [18]. Microbial VOCs have effects on various nematodes [19]. For example, the six VOCs produced by *Bacillus altitudinis* AMCC1040, namely 2,3-butanedione, acetic acid, 2-isopropoxy ethylamine, 3-methylbutyric acid, 2-methylbutyric acid, and octanoic acid, showed significant nematicidal activity against *Meloidogyne incognita* [20]. *Pseudomonas putida* isolated from the Antarctic soil produced seven VOCs, namely 1-(ethenyloxy)-octadecane, 2-undecanone, 2-nonanone, (Z)-hexen-1-ol acetate, dimethyl disulfide, 1-undecene, and 2-octanone, which were effective against *M. incognita* and *Caenorhabditis elegans* [21].

Previously, we obtained *Bacillus velezensis* GJ-7 isolates from the rhizosphere soil of *Panax notoginseng*, which displayed resistance against *Meloidogyne hapla* [22], but little is known about whether volatile organic compounds (VOCs) were produced by the strain. In the present study, we further evaluated the nematicidal activity of the volatile gas of GJ-7 in vitro and identified the VOCs using solid-phase micro-extraction-gas chromatography-mass spectrometry (SPME-GC-MS) analysis. In addition, we also evaluated their nematicidal, fumigation, and chemotaxis activities against *Meloidogyne hapla*.

## 2. Results

### 2.1. Evaluation of Nematicidal Activity of Bacillus velezensis GJ-7 VOCs

**Figure 1:**
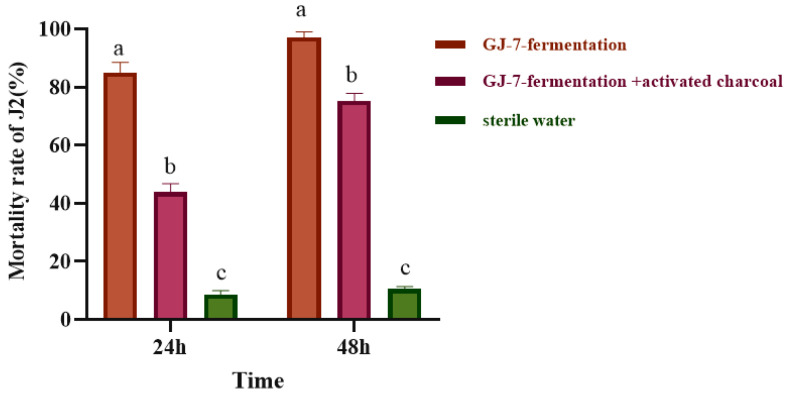
In vitro evaluation of the nematicidal activity of *B. velezensis* GJ-7 against *M. hapla* in a three-compartment Petri dish. Different lowercase letters indicate significant differences among treatments (*p* < 0.05); bars indicate the standard error of the means (*n* = 3).

We evaluated the effects of VOCs produced by *B. velezensis* GJ-7 on *M. hapla* J2s via a three-compartment Petri plate experiment (Figure 1). The results showed that the mortality rate of J2s was 85% at 24 h and 97.1% at 48 h after treatment with GJ-7-fermented VOCs. The mortality rate of J2s was 43.8% at 24 h and 75.3% at 48 h after treatment with GJ-7-fermented VOCs and activated carbon. In addition, the mortality rate of J2s treated with sterilized water (control) was 8.5% at 24 h and 10.5% at 48 h. These results suggested that the fermentation broth of the GJ-7 strain containing VOCs caused the death of nematodes, and that the adsorption of activated carbon reduced the mortality rate of nematodes caused by VOCs.

### 2.2. Identification of GJ-7 VOCs through SMPE-GC-MS Analysis

**Figure 2:**
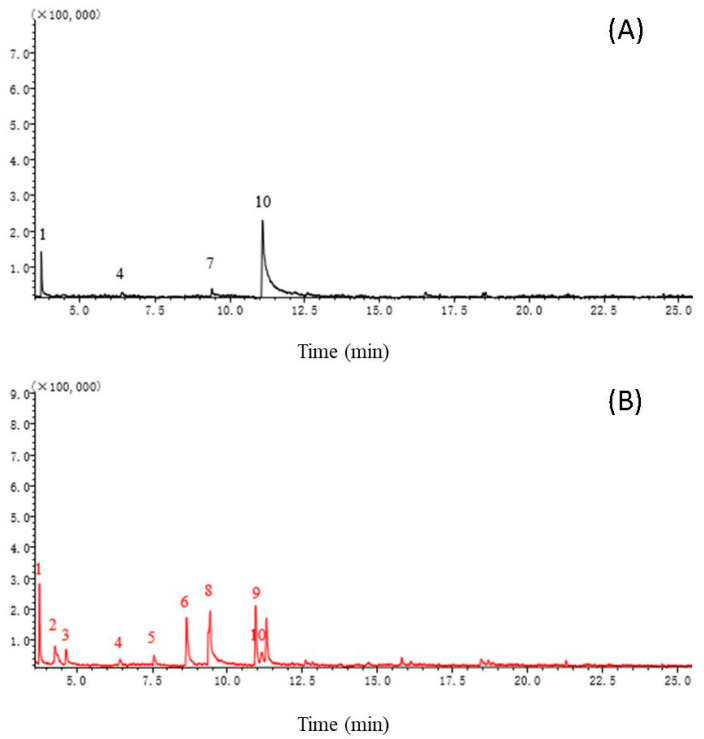
The volatiles were extracted by SPME and analyzed by GC-MS. LB medium (**A**); the GJ-7 strain fermentation broth (**B**). Peaks: (1) dimethyl-silanediol, (2) 3-methyl-1-butanol, (3) 3-methyl-2-pentanone, (4) hexamethyl-cyclotrisiloxane, (5) 5-methyl-2-hexanone, (6) 2-heptanone, (7) Oxime-, methoxy-phenyl-, (8) 2,5-dimethylpyrazine, (9) 6-methyl-2-heptanone, (10) benzaldehyde.

VOCs of the GJ-7 strain were identified based on the comparison of GC-MS results (Figure 2) with the NIST Mass Spectra database with a similarity index (SI) > 95. We identified six characteristic VOCs in the GJ-7 strain (Table 1), all of which included a diverse collection of alcohols, ketones, and pyrazines. The VOCs were selected for time durations of 0–25 min. The numbers represent peaks for different VOCs, and they are, respectively: 3-methyl-1-butanol for peak 2, 3-methyl-2-pentanone for peak 3, 5-methyl-2-hexanone for peak 5, 2-heptanone for peak 6, 2,5-dimethylpyrazine for peak 8, and 6-methyl-2-heptanone for peak 9.

### 2.3. Evaluation of Each VOC for Direct-Contact Nematicidal Activity against Meloidogyne hapla

In order to verify the direct-contact nematicidal activity of the GJ-7 strain VOCs against *M. hapla*, the pure VOCs identified through GC-MS analysis were purchased to test the contact nematicidal activity against J2s and the direct-contact inhibition activity against egg hatching. The results showed that the LC_90_ values of 3-methyl-2-pentanone, 5-methyl-2-hexanone, and 2,5-dimethylpyrazine was 10–20 μL/mL at 24 h, and the LC_90_ values of 2-heptanone and 6-methyl-2-heptanone were 6.2 and 6.4 μL/mL, respectively. In addition, 3-methyl-1-butanol was the most active, displaying LC_90_ values of 2.8 μL/mL at 24 h (Table 2).

**Figure 3:**
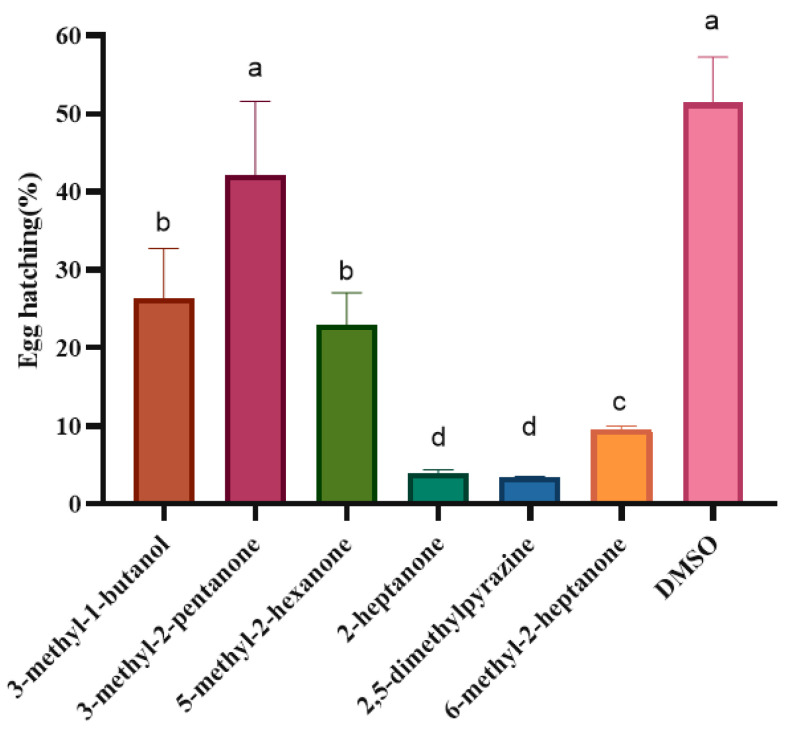
Effects of direct contact of six VOCs on the egg hatching of *M. hapla* after 7 days. Different lowercase letters indicate significant differences among treatments (*p* < 0.05); bars indicate the standard error of the means (*n* = 3).

Similarly, after continuous observation, the effects of each of the VOCs on egg hatching were recorded after 7 days of treatment. When the dilution concentration was 10 μL/mL, the hatching rate of eggs in the 3-methyl-2-pentanone treatment group was 42.08%, which represented no significant difference compared with the 51.42% of the control group (10 μL/mL DMSO). The hatching rate of the eggs treated with 3-methyl-1-butanol, 5-methyl-2-hexanone, 2-heptanone, 2,5-dimethylpyrazine and 6-methyl-2-heptanone was significantly lower than that of the control group, indicating that these five VOCs had a level of direct-contact inhibitory activity on the egg hatching of *M. hapla*. Remarkably, 2-heptanone, 2,5-dimethylpyrazine, and 6-methyl-2-heptanone had an inhibitory effect on egg hatching 2.5–7.5 times greater than other VOCs, showing strong egg hatching inhibition (Figure 3).

### 2.4. Evaluation of Each VOC for Their Fumigant Activity against Meloidogyne hapla

**Figure 4:**
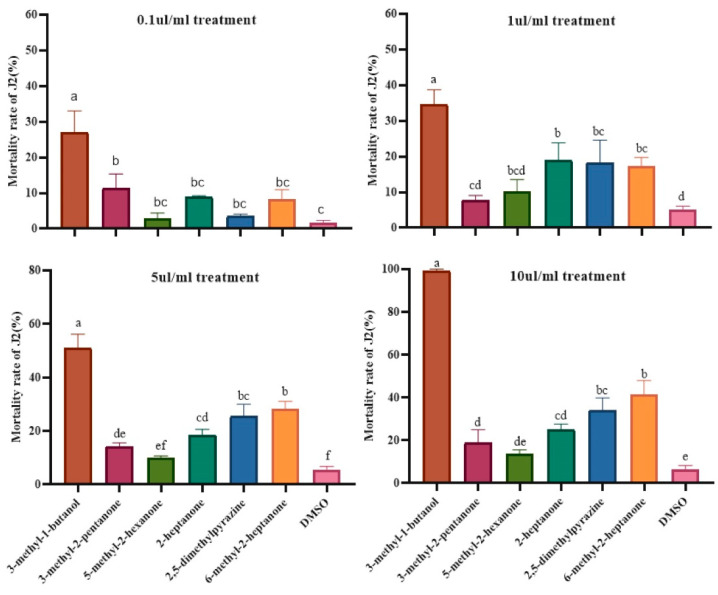
Fumigant effects of six VOCs on second stage juveniles at different concentrations. Different lowercase letters indicate significant differences among treatments (*p* < 0.05); bars indicate the standard error of the means (*n* = 3).

**Figure 5:**
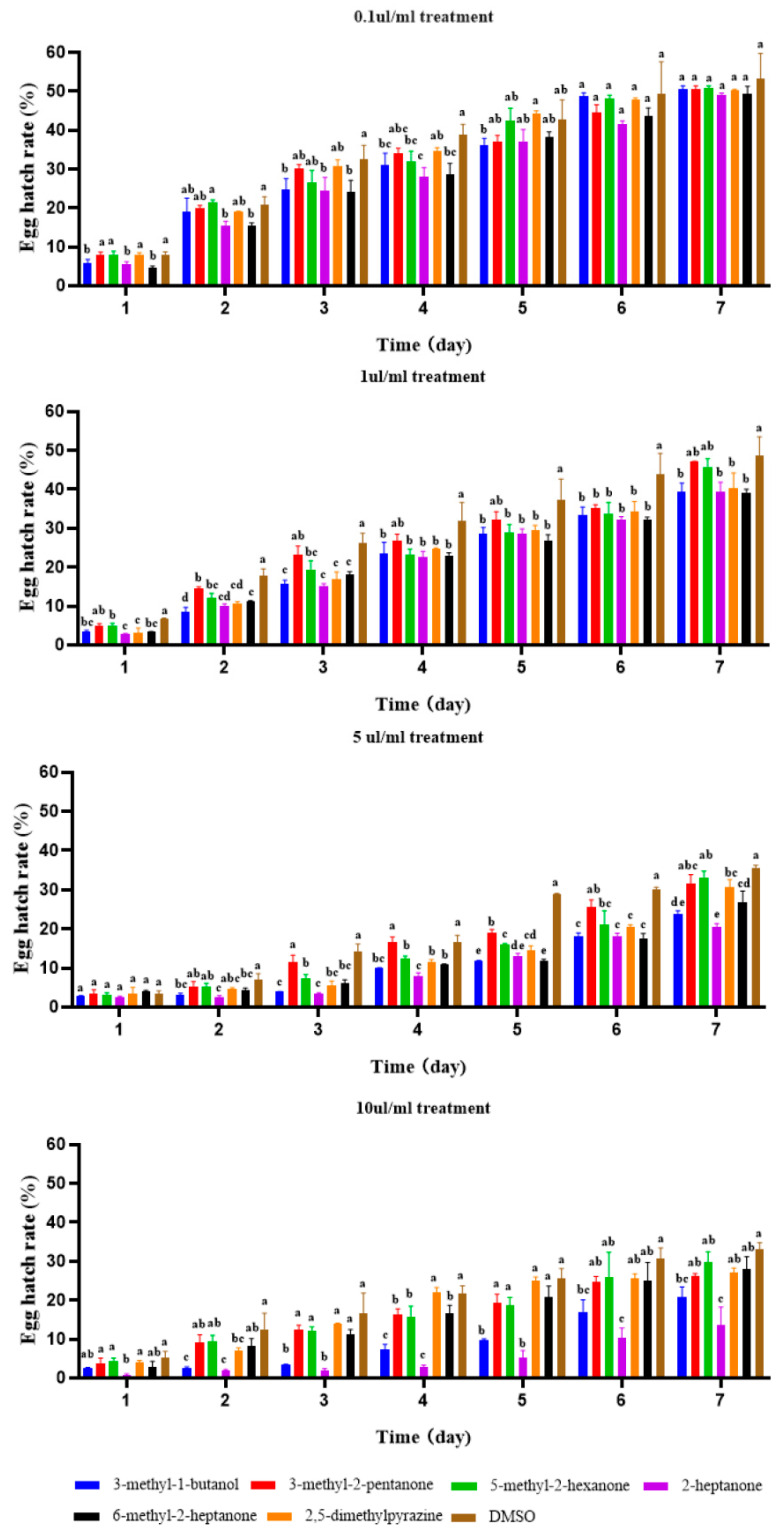
Impact of fumigation by six VOCs at different concentrations on egg hatching of root-knot nematodes after 1, 2, 3, 4, 5, 6, and 7 days of incubation. Different lowercase letters indicate significant differences among treatments (*p* < 0.05); bars indicate the standard error of the means (*n* = 3).

The fumigation activity of VOCs was evaluated by recording the mortality rate of the J2s and the egg hatching rate at the same distance from different concentrations of VOCs. The results showed that the 3-methyl-1-butanol, 5-methyl-2-hexanone, 2-heptanone, 2,5-dimethylpyrazine, and 6-methyl-2-heptanone all displayed fumigation activity against J2s, and the effect was more significant with increasing concentrations. Of these, 3-methyl-1-butanol showed particularly significant effects. When the concentration was 10 μL/mL, the mortality rate of 3-methyl-1-butanol to J2s was 99.7% (Figure 4). Additionally, through continuous observation, all VOCs at different concentrations had significant inhibitory effects on egg hatching in the first 4 days of treatment. The 3-methyl-1-butanol and 2-heptanone significantly reduced the hatchability of eggs at the concentration of 10 μL/mL, and the hatching rate was 20.8% and 13.5% after 7 days of observation, which was 32.3% and 55% lower than that of the control (Figure 5). The above results indicate that these VOCs have volatile diffusivity and can generally inhibit the hatching of eggs and have fumigation effects on J2s.

### 2.5. Chemotaxis of VOCs to Meloidogyne hapla J2s

The chemotaxis of VOCs of the GJ-7 strain is presented in Table 3. Many *M. hapla* J2s were found in the test area (A), and the six VOCs at concentrations of 0.1–100 μL/mL showed a C.I. ranging from −1 to 0 (Table 3). These results indicated that the six VOCs repelled *M. hapla* J2 juveniles. Among them, the C.I. of different concentrations of 5-methyl-2-hexanone, 2,5-dimethylpyrazine, and 6-methyl-2-heptanone showed no significant differences to the control.

In addition, with the increase in the concentration of 3-methyl-1-butanol, 3-methyl-2-pentanone, and 2-heptanone, the absolute value of the C.I. gradually increased, and the three VOCs gained a greater ability to repel nematodes. Comparing the C.I. of all six VOCs at different concentrations, we found that 3-methyl-1-butanol and 2-heptanone had the greatest effects, and their C.I. was significantly higher than those of other VOCs.

## 3. Discussion

Many metabolites produced during the growth of microorganisms and plants display nematicidal activity, so these metabolites have high potential as biological nematicides [23,24]. Among metabolites, volatile organic compounds (VOCs) have received wide-spread attention because of their potential to control RKNs and other plant pathogens (such as insects, fungi, and bacteria) [15]. Compared with non-volatile nematicidal metabolites, the advantage of volatile nematicidal metabolites is that they have good diffusion and permeability. Previous studies showed that various VOCs produced by *Bacillus* spp. (*Bacillus cereus*, *Bacillus atrophaeus*, *Bacillus altitudinis*) in soil showed significant nematicidal activity against root-knot nematodes [19,20]. Therefore, the VOCs produced by *Bacillus* spp. have great application prospects in controlling root-knot nematodes. The *B. velezensis* GJ-7 was isolated from the rhizosphere soil of *P. notoginseng*, which acts against *M. hapla* [22]. In our previous study, *B. velezensis* GJ-7 fermentation broth showed strong nematicidal activity against *M. hapla* J2s and suppressed the hatching of eggs in vitro, which means it produced metabolites against root-knot nematodes. However, little was known about whether the volatile organic compounds (VOCs) produced by *B. velezensis* GJ-7 were effective in biocontrol against *M. hapla*. Further study of the main nematicidal metabolites of *B. velezensis* GJ-7 is of great significance for the development of biological pesticides. In this study, we tested the nematicidal activity of the GJ-7 strain VOCs against *M. hapla* through a three-compartment Petri plate experiment. The results suggested that the GJ-7 strain may produce VOCs that act against *M. hapla* J2s.

Volatile organic compounds (VOCs) usually exist as complex mixtures of low molecular weight lipophilic compounds [25]. These can be alcohols, ketones, hydrocarbons, terpenes, fatty acids, or heteroatom-containing compounds [26]. So far, more than 1000 VOCs produced by more than 400 species of bacteria and fungi have been described [27,28]. The solid-phase micro-extraction gas chromatography-mass spectrometry (SPME-GC/MS) is widely used for the collection and identification of VOCs. In this method, VOCs are adsorbed by the SPME fiber from the headspace of a culture medium. The adsorbed compounds are then separated with gas chromatography and further identified with mass spectrometry. In this study, SPME-GC/MS technology was also used to analyze and identify the VOCs produced by *B. velezensis* GJ-7. A total of six characteristic VOCs were detected from the fermentation broth of the strain, which were 3-methyl-1-butanol, 3-methyl-2-pentanone, 5-methyl-2-hexanone 2-heptanone, 2,5-dimethylpyrazine and 6-methyl-2-heptanone.

**Figure 6:**
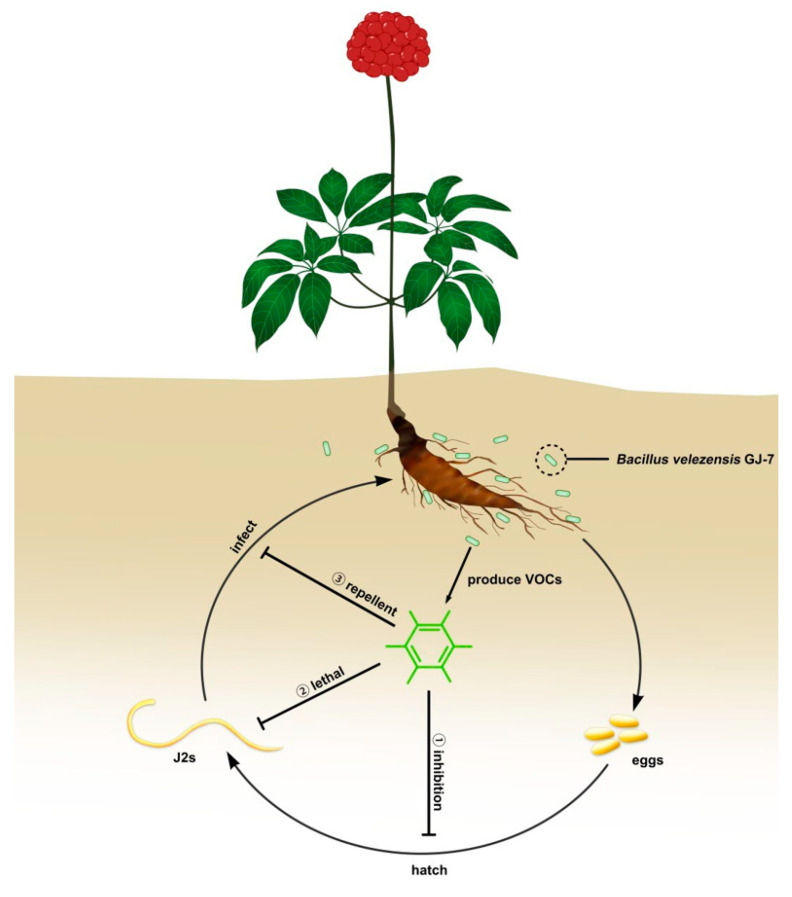
Action modes of VOCs of *Bacillus velezensis* GJ-7 against root-knot nematodes.

The nematicidal mechanism and chemical structure of bacterial VOCs are similar to those of traditional fumigants, and the VOCs can act on nematodes through direct contact, fumigation, repellent or attraction [29,30,31]. For example, the VOCs produced by *Virgibacillus dokdonensis* MCCC 1A00493 showed several activities against *M. incognita*. Acetaldehyde displays the activities of attraction, direct contact, and fumigation against root-knot nematodes, whereas ethylbenzene and 2-butanone show attraction and repellent activities, respectively [32]. *Paenibacillus polymyxa* KM2501-1 caused the 87.6% mortality of *M. incognita* in vitro and produced eleven VOCs. Of these, furfural acetone and 2-decanol were able to attract and then kill *M. incognita* through fumigation and direct contact [33]. We evaluated the way in which the VOCs of *B. velezensis* GJ-7 act on *M. hapla*, including the direct-contact nematicidal activity against the *M. hapla* J2s, the direct-contact inhibition activity against egg hatching, the fumigation activity against the *M. hapla* J2s and eggs hatching, and the chemotaxis activity against the *M. hapla* J2s. The results showed that all the six VOCs had a certain level of direct-contact inhibition and fumigation inhibition activities against J2s and the eggs of *M. hapla*. In addition, the results of chemotaxis experiment showed that all of the six volatile compounds displayed repellent activity against root-knot nematodes. Based on the above data, we predicted that the VOCs from *B. velezensis* GJ-7 have three modes to control root-knot nematode (Figure 6): ① fumigate or act directly on eggs to inhibit egg hatching; ② kill the J2s by fumigation or direct contact; ③ display repellent activity toward J2s. This research further demonstrated the mechanism of nematicidal activity of *B. velezensis* GJ-7 VOCs against *M. hapla*.

## 4. Material and Methods

### 4.1. Collection of Meloidogyne hapla Eggs and Second-Stage Juveniles

Egg masses of *M. hapla* were obtained from *P. notoginseng* plants infected with the nematodes, which were grown in the greenhouses of the Daheqiao farm of Yunnan Agricultural University (103°16′49″ E, 25°31′2″ N). The egg masses were sterilized with 1% NaOCl (sodium hypochlorite) solution for 3 min, washed three to five times with sterilized water, and the sterilized eggs were collected. In addition, the eggs were incubated in sterile water at 28 °C for 24 h to collect second-stage juveniles (J2s) [34,35].

### 4.2. Preparation of Bacillus velezensis GJ-7 Fermentation Broth

The GJ-7 strain was isolated from rhizosphere soil of *P. notoginseng* and identified as *Bacillus velezensis* via the sequence homology of the 16S rDNA. The *B. velezensis* GJ-7 strain was cultured in 100-mL flasks containing 50 mL of LB liquid medium (5 g yeast extract, 10 g tryptone and 10 g NaCl in 1 L sterilized water). The culture conditions comprised 30 °C and 220 rpm for 48 h; the cell cultures were diluted with sterilized water to a density of 1.0 × 10^8^ colony-forming units (CFU)/mL [36].

### 4.3. Nematicidal Activity of Volatiles of Bacillus velezensis GJ-7

**Figure 7:**
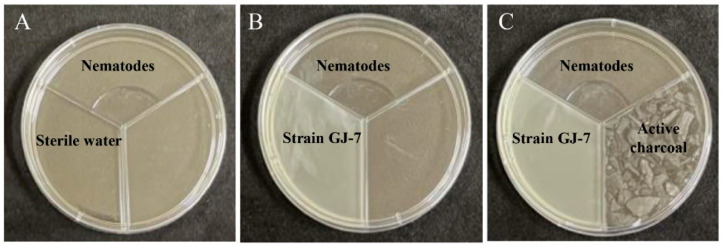
Nematicidal activity of the GJ-7 strain volatile organic compounds (VOCs) in three-compartment Petri plates (**A**–**C**).

Based on previous studies and some modifications, we tested and confirmed the nematicidal activity of the GJ-7 strain VOCs against *M. hapla* via a three-compartment Petri plate experiment in vitro [23]. A total of 5 mL of GJ-7 crude fermentation broth (1.0 × 10^8^ CFU/mL) or sterilized water (control) was added into the first compartment of the three-compartment Petri plate (diameter = 90 mm), and 200 J2s of *M. hapla* in 1 mL of sterilized water were added into the second compartment (Figure 7A,B). In the third compartment, 8 g of activated carbon was added to absorb the VOCs (Figure 7C). Then, each Petri plate was immediately wrapped in Parafilm to prevent the escape of the VOCs, and the Petri plates were incubated at 28 °C for 48 h in the dark. Each treatment consisted of three Petri plates, and the test was repeated three times independently. Mortality rates of J2s were recorded at 24 and 48 h, respectively.

Mortality rate = (Number of dead nematodes/Total nematodes) × 100%(1)

### 4.4. Detection and Identification of VOCs from Bacillus velezensis GJ-7

The solid-phase micro-extraction (SPME) method was used to collect the VOCs of the GJ-7 strain of *B. velezensis* [37,38]. The GJ-7 strain fermentation broth (1.0 × 10^8^ CFU/mL) was prepared as described above, and 5 mL fermentation liquid was placed into 15 mL headspace vials. The polydimethylsiloxane/divinylbenzene (PDMS/DVB) fiber used for the SPME was first preconditioned with helium at 250 °C for 15 min, and then exposed to the headspace volatiles of the GJ-7 fermentation broth and equilibrated at 60 °C for 1 h. The VOCs from the 5 mL LB medium were used as controls. After the collection, gas chromatography-mass spectrometry (GC-MS) analysis was carried out using the gas chromatograph coupled with a mass selective detector. The fiber was inserted into the injection port of a gas chromatograph and desorbed at 250 °C for 5 min. The chromatographic separation was performed on a HP-5MS capillary column (30 m length; 0.25 mm internal diameter; 0.25 μm particle size) and helium was used as the carrier gas. The column temperature was held at 40 °C for 2 min, increased to 180 °C at a rate of 4 °C/min, then increased further to 240 °C at a rate of 5 °C/min and held for 6 min. The mass spectrometer was operated in an electron ionization mode of 70 eV and frequently scanned at 35 to 550 *m*/*z*. The identification of volatile compounds was confirmed by comparing their spectra and retention times with data from the National Institute of Standards and Technology (NIST). The possible volatiles identified through GC-MS analysis were bought from the Rhawn company, Shanghai China.

### 4.5. Direct-Contact Nematicidal Activity of Commercial VOCs against Meloidogyne hapla

The direct-contact nematicidal activity of commercial VOCs was tested against *Meloidogyne hapla* J2 with different concentrations in a 24-well plate experiment, and the 90% lethal concentration (LC90) values were calculated. The pure compounds were diluted with indimethyl sulfoxide, then added to sterile water for further dilution. The compound solutions (1 mL) with different concentrations were added to 24-well tissue culture plates, and then the suspension of approximately 100 J2 juveniles was added to each well. The plates were covered with plastic lids and sealed with Parafilm to avoid evaporation, and then plates were kept at 25 °C in the dark. After 24 h and 48 h of exposure, nematode mortality was recorded under an inverted microscope. In addition, similar to the J2 mortality test described above, the effects of compound solutions with different concentrations on egg hatching were tested in 24-well plates. Samples of 1 mL of compound solutions at different concentrations were mixed with approximately 100 eggs in separate wells. The 24 well plates were incubated in the chamber at 28 °C for 7 days, and the number of J2 hatched in each well was recorded [39,40]. The sterile distilled water containing dimethyl sulfoxide served as controls (DMSO). The experiments were performed three times, and every treatment was replicated three times.

Hatching rate = (the number of hatched J2/total eggs) × 100%(2)

### 4.6. Fumigant Activity of Commercial VOCs against Meloidogyne hapla

To test the fumigant activity of commercial VOCs against *Meloidogyne hapla*, the commercial compound was diluted to solutions of 0.1, 1, 5, and 10 μL/mL. The commercial VOCs with different concentrations were added to one well in 24-well tissue culture plates, and then 100 nematode J2 juvenile or 100 eggs were placed in suspension (1 mL) into the four surrounding wells. Plate lids were sealed with Parafilm and plates were incubated in the dark at 28 °C [41]. After 24 h of exposure, the mortality of J2s was recorded in response to the fumigant activity of the VOCs in the adjacent wells. In addition, the numbers of J2 hatchings were counted after 1, 2, 3, 4, 5, 6 and 7 days of exposure under an inverted microscope, and hatch rate was measured. The experiments were performed three times, and every treatment was replicated three times.

### 4.7. Chemotaxis Activity of VOCs to the Meloidogyne hapla J2s

**Figure 8:**
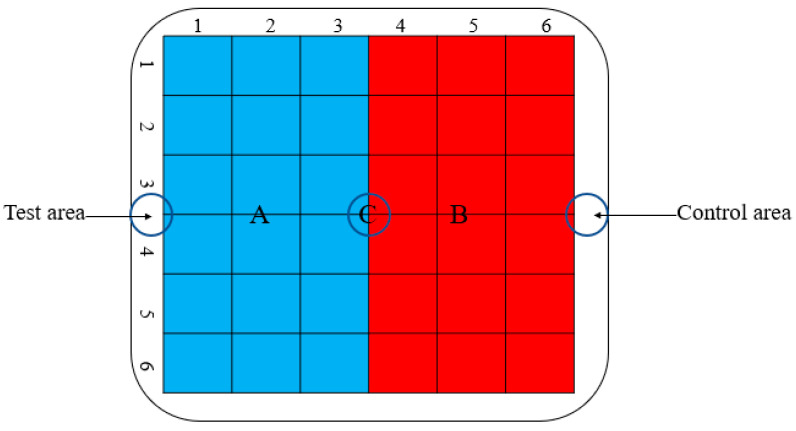
Schematic diagram of the chemotaxis test in square Petri dishes. A: test area; B: control area; Blue represents area A; Red represents area B.

The chemotaxis activity test was carried out in square Petri dishes (width = 10 cm) containing 2% water agar (WA), as described previously [42], with some modifications. Firstly, filter paper discs (5 mm) soaked with volatile compound solutions of different concentrations were placed on the test area (area A) of square Petri dishes, and filter paper discs soaked with sterile distilled water containing dimethyl sulfoxide were placed on the control area (area B) of square Petri dishes as a control (Figure 8). Subsequently, approximately 150 *M. hapla* J2s in 10 μL of sterilized water were inoculated at the center (area C) of the Petri dishes and cultured in the dark at 25 °C for 12 h. The chemotaxis index (C.I.) was calculated by recording the number of J2s in areas A and B under the anatomical microscope [43]. The experiments were performed three times, and every treatment was replicated three times.

C.I. = (the number of nematodes in the test area − the number of nematodes in the control area)/(the total number of nematodes)(3)

If 0 < C.I. < 1, the *M. hapla* J2s were attracted by the tested sample; if −1 < C.I. < 0, the tested sample repelled *M. hapla* J2s; and if C.I. = 0, the sample had no effect on the *M. hapla* J2s.

### 4.8. Data Analysis and Statistics

All the data were recorded and organized in Microsoft Office Excel (2010). The mean ± standard deviation (SD) values were calculated, and a one-way analysis of variance (ANOVA) was carried out in DPS. Statistically significant differences were determined using Duncan’s multiple range test at *p* < 0.05.

## 5. Conclusions

In conclusion, our study confirmed that the volatile organic compounds (VOCs) produced by *B. velezensis* GJ-7 had significant nematicidal activity. A total of six GJ-7 strain VOCs were identified by SPME-GC-MS, including alcohols, ketones and pyrazines. These VOCs showed strong direct-contact nematicidal activity, fumigant activity, and repellent activity against *M. hapla*. The study provides new insight into the mechanisms of *Bacillus* VOCs against root-knot nematodes.

## Figures and Tables

**Table 1:** GC/MS analysis of strain GJ-7 fermentation broth.

Compound	RT (min)	SI	Molecular Weight	Peak Area (%)	Peak Number
3-methyl-1-butanol	4.29	96	88.15	4.44	2
3-methyl-2-pentanone	4.665	97	100.16	3.56	3
5-methyl-2-hexanone	7.565	96	114.19	2.46	5
2-heptanone	8.65	98	114.19	14.49	6
2,5-dimethylpyrazine	9.375	95	108.14	16.28	8
6-methyl-2-heptanone	10.95	96	128.21	15.56	9

RT, retention time; SI, similarity index.

**Table 2:** Nematicidal activity of six commercial VOCs against second stage juveniles.

Compound	24 h	48 h
	LC_90_ (μL/mL)	LC_90_ (μL/mL)
3-methyl-1-butanol	2.8	2.7
3-methyl-2-pentanone	20	18
5-methyl-2-hexanone	20	17
2-heptanone	6.2	6.1
2,5-dimethylpyrazine	12.7	10.8
6-methyl-2-heptanone	6.4	6.3

**Table 3:** Chemotaxis index values of commercial VOCs toward *M. hapla*. Different lowercase letters indicate significant differences among different concentrations of the same VOC. (*n* = 3).

Compound	Chemotaxis Index (C.I.)			
	0.1 μL/mL	1 μL/mL	10 μL/mL	100 μL/mL
3-methyl-1-butanol	−0.044 ± 0.0006 b	−0.274 ± 0.025 a	−0.284 ± 0.042 a	−0.299 ± 0.037 a
3-methyl-2-pentanone	−0.019 ± 0.006 b	−0.033 ± 0.015 b	−0.103 ± 0.007 a	−0.112 ± 0.018 a
5-methyl-2-hexanone	−0.035 ± 0.007 b	−0.057 ± 0.028 b	−0.095 ± 0.012 ab	−0.148 ± 0.037 a
2-heptanone	−0.111 ± 0.017 b	−0.216 ± 0.022 a	−0.270 ± 0.034 a	−0.258 ± 0.003 a
2,5-dimethylpyrazine	−0.064 ± 0.017 a	−0.048 ± 0.003 a	−0.052 ± 0.013 a	−0.079 ± 0.003 a
6-methyl-2-heptanone	−0.022 ± 0.015 b	−0.023 ± 0.018 b	−0.039 ± 0.023 b	−0.120 ± 0.036 a

## Data Availability

Not applicable.
